# Sounding It Out: Auditory Stimulation and Overnight Memory Processing

**DOI:** 10.1007/s40675-021-00207-0

**Published:** 2021-07-16

**Authors:** Marcus O. Harrington, Scott A. Cairney

**Affiliations:** 1 Department of Psychology, University of York, York, UK; 2 York Biomedical Research Institute, University of York, York, UK

**Keywords:** Closed-loop stimulation, Slow-wave sleep, Slow oscillations, Sleep spindles, Rapid eye movement sleep, Memory consolidation

## Abstract

**Purpose of Review:**

Auditory stimulation is a technique that can enhance neural oscillations linked to overnight memory consolidation. In this review, we evaluate the impacts of auditory stimulation on the neural oscillations of sleep and associated memory processes in a variety of populations.

**Recent Findings:**

Cortical EEG recordings of slow-wave sleep (SWS) are characterised by two cardinal oscillations: slow oscillations (SOs) and sleep spindles. Auditory stimulation delivered in SWS enhances SOs and phase-coupled spindle activity in healthy children and adults, children with ADHD, adults with mild cognitive impairment and patients with major depression. Under certain conditions, auditory stimulation bolsters the benefits of SWS for memory consolidation, although further work is required to fully understand the factors affecting stimulation-related memory gains. Recent work has turned to rapid eye movement (REM) sleep, demonstrating that auditory stimulation can be used to manipulate REM sleep theta oscillations.

**Summary:**

Auditory stimulation enhances oscillations linked to overnight memory processing and shows promise as a technique for enhancing the memory benefits of sleep.

## Introduction

After a longstanding debate, it is now generally accepted that memory consolidation is supported by sleep. Contemporary models of sleep-associated consolidation posit that memories of recent experiences are actively strengthened during the deepest stage of non-rapid eye movement (NREM) sleep: slow-wave sleep (SWS) [1, 2]. Cortical EEG recordings of SWS are characterised by <1 Hz slow oscillations (SOs), which emerge in neocortical regions and reflect widespread synchronous activity alternating between up-states of neuronal excitation and down-states of neuronal silence. Crucially, SOs are thought to synchronise fast thalamo-cortical spindles (~12–16 Hz) and hippocampal ripples (80–100 Hz in humans) to their depolarising up-states, and, in doing so, drive the repeated reactivation of hippocampal memory representations [1, 2].

An abundance of studies have linked SWS and SOs to memory consolidation in humans [3–10]. Notwithstanding the obvious importance of this work, many early reports relied on correlational findings, and thus lacked causal evidence of SOs actively contributing to overnight memory processing. The first efforts to address this gap used electrical stimulation to experimentally induce SOs during SWS, which, relative to a control condition, improved memory retention [11, 12]. Importantly, however, these electrical stimulation studies artificially imposed SO rhythms on the brain that were not synchronised to the brain’s endogenous oscillatory activity, potentially limiting the benefits of SO induction on sleep-associated memory processing.

In their seminal study, Ngo and colleagues [13] used an innovative auditory stimulation method to enhance the brain’s own endogenous rhythm and, consequently, bolster the memory benefits of SWS (see Box 1). This work paved the way for more auditory stimulation studies that aimed to elucidate the link between NREM sleep oscillations and offline memory processing. In this review, we provide an overview of the literature in this important and rapidly developing field. We first describe the variety of ways in which researchers have modified the parameters of auditory stimulation, and the impact that these modifications have had on the neural oscillations of sleep and associated memory processes. Next, we outline how the effects of stimulation vary according to the demographics of the study participants, focusing on different age groups and clinical samples. Finally, we discuss the behaviourally observable effects of stimulation in different memory domains.

## Box 1

**Fig. 1:**
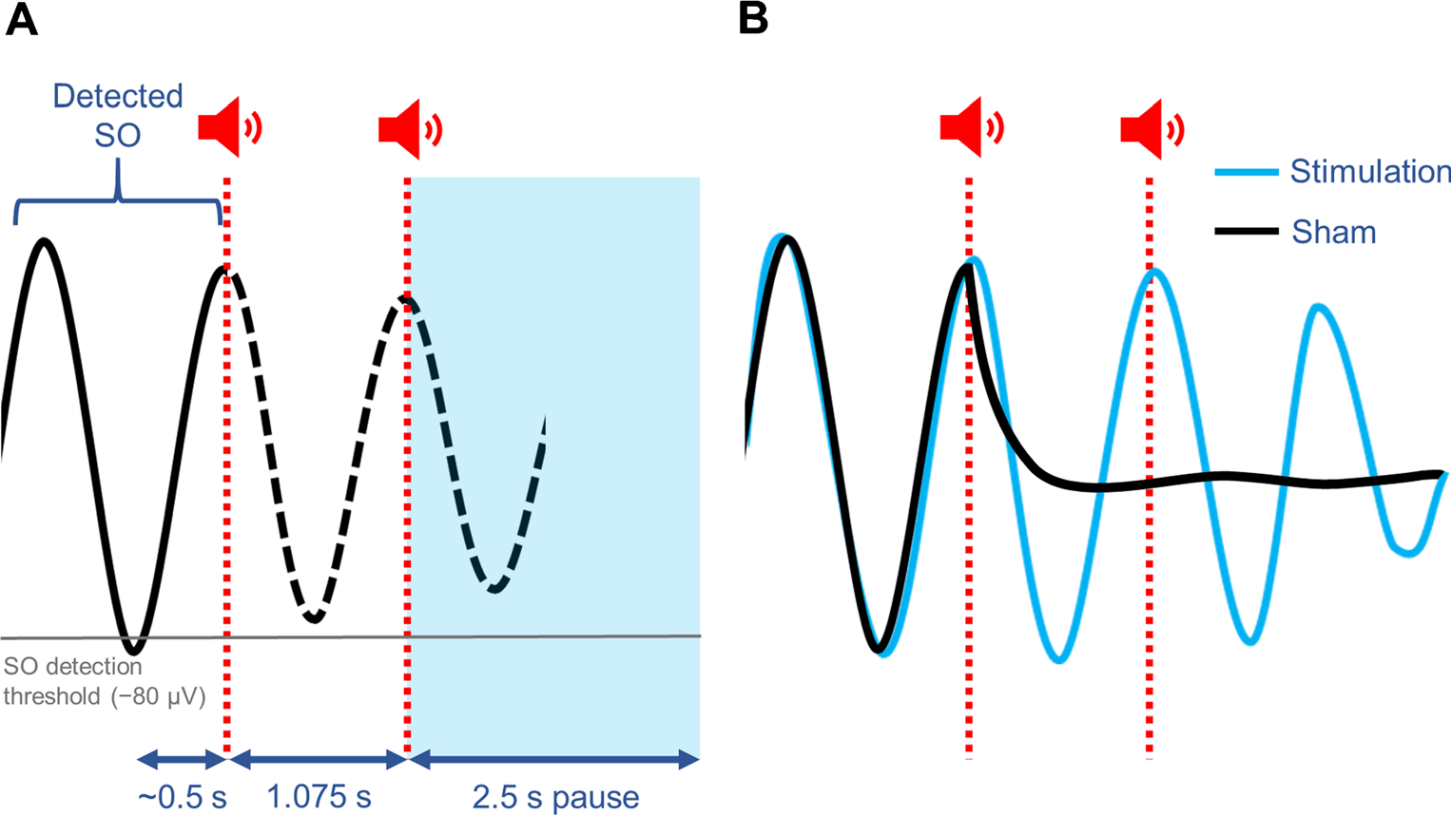
Example of the auditory stimulation protocol used by Ngo and colleagues [13], and its effects on the SO rhythm. **A.** Following detection of a supra-threshold SO down-state (−80 μV; grey line), two 50 ms pulses of pink noise (clicks) are delivered in phase with the two subsequent SO up-states (red lines). The first click occurs during the predicted SO up-state, based on the average delay between the SO negative and positive peak (~0.5 s; obtained for each participant during a prior adaptation night), and the second after a 1.075 s interval corresponding to the typical duration of a full SO cycle. In the sham condition, corresponding time points of would-be stimulation events are marked but no clicks are delivered. The detection routine is paused for 2.5 s after the second click. **B.** Relative to the sham condition, stimulation typically enhances the SO rhythm, evoking a ‘train’ of three SOs

Ngo and colleagues [13] delivered auditory stimulation during SWS in synchrony with the brain’s own endogenous rhythm. Healthy young adults each participated in two conditions: stimulation and sham. In the stimulation condition, frontal EEG activity was recorded in real time and, following algorithmic detection of a supra-threshold SO down-state, two 50 ms pulses of pink noise (clicks) were delivered in phase with the two subsequent SO up-states (see example in Fig. 1A). Timing of the clicks varied according to the temporal characteristics of each participant’s SOs (i.e. the average time between the negative and positive SO peaks), thereby ensuring precise, phase-locked stimulation. In the sham condition, would-be stimulation events were marked but no clicks were delivered. Auditory stimulation was found to enhance the SO rhythm. Specifically, stimulation induced ‘trains’ of three successive SO cycles (as compared to the individual SOs observed in the sham condition, see example in Fig. 1B), increased the amplitude of SO cycles and amplified phase-coupled fast spindle activity during SO up-states. Moreover, stimulation (vs. sham) led to a sizeable improvement in overnight memory retention, supporting the view that SOs play a causal role in offline consolidation. Auditory stimulation out of phase with the ongoing SO rhythm did not enhance SO activity or improve memory performance compared to sham.

## Stimulation Parameters

### Number of Clicks

Given the observed effects of auditory stimulation with two clicks, an obvious question is whether additional clicks lead to further enhancement of oscillatory activity and sleep-associated consolidation.

Ngo and colleagues [14] addressed this question in a follow-up study by comparing their original two-click protocol to a ‘driving stimulation’ protocol, which delivered up to four consecutive clicks (also during SO up-states). Driving stimulation (vs. sham) prolonged SO trains, amplified phase-locked fast spindle activity and improved overnight retention; but to no greater extent than that afforded by two-click stimulation. Similar findings have emerged from more recent work using five-click stimulation protocols [15–17]; whereas stimulation enhanced the SO rhythm and improved next-day recall, the amplitude of the SOs induced by the clicks typically declined after the third SO cycle.

Hence, the research to date suggests that delivering more than two clicks does not confer any additional enhancement to SO activity or overnight memory processing. Because enhancing the SO rhythm and synchronised excitability carries an increased risk of seizure-like activity, it has been suggested that limitations in the sleeping brain’s responsiveness to auditory stimulation might reflect the presence of a protective mechanism that prevents the development of hypersynchronicity during SO activity [14, 18].

Interestingly, a number of studies using only a single auditory pulse per stimulation event have observed enhancements in NREM oscillatory activity [19••, 20–22] and overnight retention [22]. Whether one-click stimulation is as effective as two-click stimulation, however, has yet to be established.

### Closed-loop Versus Open-loop Methods

The vast majority of auditory stimulation studies have used the closed-loop method; that is, utilising the ongoing EEG activity to deliver auditory stimuli in synchrony with the brain’s endogenous rhythm [13, 14, 16, 17, 19, 21–31, 32]. One study, however, used an open-loop protocol in which the ongoing EEG data had no bearing on the timing of stimulus delivery [33]. For each stimulation event, an initial click was delivered without regard to SO phase. Two further clicks were then delivered at times chosen to maximise their probability of coinciding with the up-states of the SOs evoked by the first click. The interval between the first and second clicks was based on the average duration of a full SO cycle for the respective participant (~0.9 s; obtained in a prior adaptation night), and the interval between the second and third clicks was fixed at 1.075 s.

Open-loop stimulation, relative to sham, evoked a train of SOs and amplified fast spindle activity during the up-state of the first SO cycle. Importantly, however, stimulation led to an overall decrease in fast spindle power across the entire stimulation period, and had no impact on memory retention when compared to the sham condition. Hence, despite clearly influencing oscillatory activity in SWS, open-loop stimulation might be ineffective at enhancing overnight consolidation. Further work is of course required to build on this single study.

### Continuous Stimulation

Other work has employed an auditory stimulation method that we refer to here as continuous stimulation [34]. Throughout the first 90 min of an overnight sleep opportunity, single clicks were delivered continuously at a frequency of 0.8 Hz (corresponding to the approximate frequency of SOs). As compared to sham, stimulation increased SO and fast spindle power during NREM sleep. However, because a memory test was not included in the study protocol, it was not possible to establish whether these stimulation-induced changes in oscillatory activity led to improvements in overnight consolidation.

### Timing of Stimulation

Because the cortical response to auditory stimulation varies according to the timing of stimulus delivery [13, 33], a recent study aimed to determine the SO phase at which clicks maximally augment oscillatory activity during SWS [26•]. Relative to sham, applying clicks at any point during the SO up-state increased the amplitude of subsequent SOs and increased the likelihood of detecting a phase-coupled sleep spindle. However, the amplitudes of stimulation-induced SOs and spindles were maximal when clicks were delivered close to the SO peak, suggesting that optimization of SO stimulation depends critically on precise timing of stimulus delivery.

### Target Oscillation: Sleep Spindles

Given their putative function in overnight consolidation, researchers have utilised auditory stimulation to directly target sleep spindles.

In the first study of its kind, oscillating white noise was delivered in 2 s bursts at regular intervals throughout stage two sleep and SWS [35]. The sounds were delivered at one of three frequencies: a slow spindle-mimicking frequency of 12 Hz, a fast spindle-mimicking frequency of 15 Hz, or a control frequency of 50 Hz. Whereas 50 Hz stimulation had no effect on spindle activity, 12 Hz and 15 Hz stimulation increased slow and fast spindle densities, respectively. The evoked spindles were comparable to endogenously generated spindles in both duration and topography, suggesting that spindle stimulation might augment offline memory processing. However, because the study protocol did not include a memory test, the effects of spindle stimulation on sleep-associated consolidation could not be assessed.

In a later study, application of a rapid sequence of clicks (~15 Hz) during SO up-states had no immediate impact on spindle activity [25]. Instead, stimulation evoked an additional SO which was accompanied by an increase in phase-coupled spindle power. This rapid click stimulation did not influence memory retention, as compared to sham. In other works, delivery of single clicks upon algorithmic detection of sleep spindles evoked a single SO, increased both delta (1–4 Hz) and theta (4–8 Hz) power and improved performance in a procedural learning task [36•].

### Target Oscillation: REM Sleep Theta

Theta oscillations (~3–7 Hz) are a prominent feature of the REM sleep EEG, and were directly targeted in a recent study using a protocol modelled on the principles of auditory closed-loop stimulation [37••]. Upon algorithmic detection of two supra-threshold theta cycles, oscillating white noise was delivered at 5 Hz, corresponding to the approximate frequency of endogenous theta waves. Relative to sham, stimulation evoked a rapid increase in theta power, which was immediately followed by a prolonged period of theta suppression, and a prolonged increase in 10–30 Hz beta power. Stimulation had no impact on overnight memory retention.

## Participant Demographics

### Older Adults

Because the vast majority of auditory stimulation studies have tested healthy young adults (typically aged 18–30 years), we focus here on the effects of stimulation in older adults.

Mirroring findings in young people, a study that delivered five-click, closed-loop stimulation to adults aged 60 to 84 years found that stimulation prolonged SO trains, amplified phase-coupled spindle activity and improved overnight memory retention, as compared to sham [29]. More recently, however, a direct comparison of the effects of auditory SO stimulation in young and older adults (aged 49–63 years) revealed a marked reduction in SO amplitude and phase-coupled spindle activity in older people [30••]. Correspondingly, the memory benefits of stimulation observed in young adults were absent in the older individuals. Other studies paint a similar picture. In healthy middle-aged men (aged 35–48 years), auditory stimulation increased 0.5–4 Hz delta power across the entire night but had no impact on memory retention [31]. Likewise, stimulation applied to older adults with mild cognitive impairment (aged 62–86 years) amplified SO power, but had no effect on overnight consolidation [16].

In sum, these findings point to an age-related decline in the effectiveness of auditory stimulation. Normal ageing is associated with a reduction in the density and amplitude of SOs, and a decoupling of SOs and spindles [38–40]. These changes reflect a deterioration of synchronised firing in large neuronal populations. It has been suggested that diminished responsivity to stimulation in older adults could be the result of decreased cortical capability to group large neural populations into synchronised activity in response to peripheral stimuli [26•]. The failure of some studies to detect a benefit of auditory stimulation on memory processing in older adults, despite augmentation of SOs and spindles, offers support to the ‘functional weakening’ hypothesis [41]. According to this view, bolstering sleep in older adults is unlikely to benefit memory because many of the neurobiological mechanisms that are necessary for overnight consolidation are otherwise impaired (e.g. because of neural atrophy that occurs in normal ageing) [42]. Understanding how the changes in brain morphology that accompany normal ageing influence the oscillatory and mnemonic impacts of auditory stimulation will be an important challenge for future research.

### Children

By the age of 12 years, children typically achieve almost twice the amount of SWS as adults [43], and can thus provide important insights into overnight consolidation processes mediated by SOs. A single study to date has applied auditory stimulation to typically developing children aged 8–12 years, and children with attention deficit hyperactivity disorder (ADHD) of the same ages [23•]. In both groups, a two-click closed-loop stimulation protocol evoked trains of three SO cycles, mirroring findings in healthy adults. Stimulation also improved memory recall relative to the sham condition. Whether the efficacy of auditory stimulation differs between children and adults has yet to be established.

### Patients with Major Depression

Sleep disturbances are a common feature of nearly all psychiatric conditions [44, 45]. Whether and how auditory stimulation affects slow oscillatory activity and associated cognitive functions among individuals with psychiatric disorders are important questions for understanding the mechanisms of disease and potential targets for therapeutic intervention. In a recent study [27], auditory stimulation (vs. sham) applied to adults with major depression increased delta (0.5–2.5 Hz) and beta (16–25 Hz) power, but decreased oscillatory activity in the slow spindle range (12.5−14.5 Hz). The impacts of stimulation on memory performance were not assessed.

## Memory Domains

Since the seminal findings of Ngo and colleagues [13], a large number of studies have assessed the memory effects of auditory stimulation across a variety of memory domains. These studies are summarised in Table 1, organised by memory domain and associated task.

**Table 1:** Studies investigating the memory effects of auditory stimulation in sleep, organised by memory domain and task

Memory domain	Task
Declarative memory	Paired associates learning: related word pairs [13, 14, 16, 17, 19••, 22, 25, 29, 30••, 33, 36•]
	Paired associated learning: unrelated word pairs [19••, 23•, 31, 32]
	Paired associates learning: face-name associations [22]
	Picture recognition task (encoding after stimulation) [24, 30••]
	Spatial navigation task [19••]
Motor skills	Finger tapping [22, 30••, 36•]
	Serial reaction time task [23•]
Emotional memory	Picture recognition [22, 37]
Working memory	N-back task [23•]

### Declarative Memory

Given that SOs are thought to drive the reactivation of memory representations in the hippocampus, most auditory stimulation studies have assessed overnight changes in hippocampus-dependent declarative memories.

Memory for semantically related word pairs (e.g. pan-hob) typically benefits from closed-loop stimulation [13, 14, 17, 22, 29] (also see: [16, 19••, 30]), but not open-loop stimulation [33], or stimulation directly targeting sleep spindles [25, 36]. By contrast, auditory closed-loop stimulation does not seem to improve memory for pairs of stimuli with no intrinsic connection, such as semantically unrelated word pairs (e.g. pan-car) or face-name pairs [19••, 22, 31, 32]. A notable exception was observed in a recent study in children, where stimulation improved retention of unrelated word pairs associated with a monetary reward, but not unrewarded word pairs [23•]. Hence, the findings to date suggest that auditory stimulation benefits the consolidation of paired associates that are consistent with pre-existing knowledge, or of personal value to the individual.

A single study has investigated the effect of auditory stimulation on visuospatial declarative memory in healthy young adults [19••]. Here, stimulation applied during a daytime nap had no impact on navigation speed in a virtual spatial navigation task.

Because sleep plays an important role in new learning [46–48], other works have tested the hypothesis that auditory stimulation improves encoding capabilities in declarative memory. In a recent study in healthy adults, the amplitude of stimulation-evoked SOs was correlated with hippocampal activation during picture encoding, and better performance in a subsequent recognition test [24]. Relative to the sham condition, however, there was no overall benefit of stimulation for learning. A subsequent study also failed to observe any benefit of auditory stimulation on picture encoding capacities in middle-aged and older adults [30••].

### Motor Skills

Research examining the effects of auditory stimulation on procedural motor skills has produced mixed results. In one study, auditory stimulation directly targeting sleep spindles (vs. sham) improved performance on a finger tapping task, such that healthy young adults were faster to type sequences of digits that were learned prior to sleep [36•]. Similarly, relative to sham, auditory SO stimulation improved sequence learning in children with ADHD [23•]. However, other studies have failed to observe a benefit of SO stimulation on finger tapping performance in healthy young adults [22] or middle-aged and older adults [30••].

### Emotional Memory

Theta oscillations during REM sleep have been linked to the consolidation of emotional memories [49, 50]. In recent work, however, manipulation of theta activity via auditory stimulation in REM sleep had no impact on the retention of negative or neutral images [37••]. Likewise, auditory stimulation during SWS, which has also been implicated in affective memory processing [51, 52], had no impact on memory for aversive pictures [22].

### Working Memory

Just one study to date has examined the effect of auditory stimulation on working memory [23•]. In children with ADHD, SO stimulation (vs. sham) improved performance in a n-back task, such that they were faster to correctly identify whether or not patterns that they were shown had appeared in recently preceding trials.

## Conclusion

Auditory stimulation is a powerful tool for inducing, augmenting and modifying the neural oscillations of sleep. Delivering auditory stimuli during SO up-states reliably enhances the SO rhythm and boosts phase-coupled spindle activity. Older adults appear to be less receptive to auditory stimulation than young adults—possibly because of age-related changes in brain morphology. Single studies have found that SO stimulation modulates neural rhythms in typically developing children, children with ADHD, adults with major depression and older adults with mild cognitive impairment. Multiple experiments have observed a significant benefit of auditory stimulation on memory retention. However, the effect of stimulation on memory seems to vary according to the type of learning material and the memory system under investigation.

Despite significant advances in our understanding of the effects that auditory stimulation can have on electrophysiology, little is known about *how* these effects occur. It has long been known that delivering auditory stimuli during NREM sleep evokes K-complexes, which are comparable to SOs in both appearance and generating mechanisms [53]. Evoked K-complexes are believed to preserve sleep continuity in the face of external stimuli that would otherwise fragment sleep by triggering arousal responses [54]. Although speculative, one possibility is that auditory stimulation exploits this adaptive K-complex response to artificially bolster overnight consolidation. Specifically, when stimulation is delivered in-phase with an SO up-state, the evoked K-complex has an additive effect on the subsequent SO cycle, which increases its amplitude. Although endogenous SOs often occur in trains, they can occur as singular events [14]. For these solitary SOs, in-phase stimulation could induce a K-complex after the detected SO cycle, giving the appearance of an SO train.

Our knowledge of how auditory stimulation benefits overnight consolidation is also somewhat superficial. Presumably, by enhancing SOs and phase-coupled spindle activity─which putatively drive reactivations of hippocampal memory representations [1, 2]─auditory stimulation increases the frequency and/or efficacy of reactivation events.

An important challenge for future research will be to elucidate the mechanisms by which acoustic stimuli boost NREM sleep oscillations, and how boosting these oscillations aids overnight consolidation. Moreover, future studies should address how receptiveness to auditory stimulation is affected by changes in brain morphology that accompany normal ageing. Such investigations could offer deeper insights into why sleep and memory decline in old age, and could also inform our understanding of neurodegenerative disorders such as Alzheimer’s disease. Finally, further experiments are required to explore the behavioural effects of enhancing NREM sleep spindles or REM sleep theta oscillations.

In sum, auditory stimulation has had a major impact on sleep and memory research, having helped to establish a causal role for NREM oscillations in mnemonic processing. The large gaps in the existing literature indicate that there is still a lot more that we can learn by using auditory stimulation techniques.
